# Correction to “Clinical Characteristics of Patients With Enlarged Ventricles and Cognitive Impairment (EVCI): Case Series”

**DOI:** 10.1002/npr2.70058

**Published:** 2025-09-15

Y. Yasuda, S. Ito, J. Matsumoto, et al., “Clinical Characteristics of Patients With Enlarged Ventricles and Cognitive Impairment (EVCI): Case Series,” *Neuropsychopharmacology Reports* 45, no. 3 (2025): e70029.

In the originally published article, there were errors in the gender description of Patient 4 in the main text. In the text description of Patient No. 4, the gender is incorrectly stated as female, whereas in Table 1, the correct gender (male) is listed correctly.

In paragraph 5 of the “Case Presentation” section, the text “Patient 4 was a 23‐year‐old female with schizophrenia. She had no specific developmental abnormalities. She has a cleft palate and had surgery at age 10. She has typical characteristics of schizophrenia with hallucinations and delusions at the age of 18 and has no history of hospitalization.” was incorrect. This should be corrected to “Patient 4 was a 23‐year‐old man with schizophrenia. He had no specific developmental abnormalities. He has a cleft palate and had surgery at age 10. He has typical characteristics of schizophrenia with hallucinations and delusions at the age of 18 and has no history of hospitalization.”

In paragraph 1 of the “Discussion” section, the text “On the other hand, the 29‐year‐old man (Patient 2) and a 23‐year‐old woman (Patient 4) had none of the risk factors.” was incorrect. This should be corrected to “On the other hand, the 29‐year‐old man (Patient 2) and a 23‐year‐old man (Patient 4) had none of the risk factors.”

We apologize for these errors.

